# Risk factors for hemodynamically significant patent ductus arteriosus and ibuprofen treatment failure in premature twins: a retrospective case-control study

**DOI:** 10.3389/fcvm.2025.1687361

**Published:** 2026-01-12

**Authors:** Bo Gao, Yixue Zhao, Huixian Li, Yue Du, Yucan Wu, Guijun Chen, Ping Wang

**Affiliations:** 1 Department of Neonatology, Guangzhou Women and Children's Medical Center, Guangzhou Medical University, Guangzhou, China; 2 The Sixth Affiliated Hospital, Sun Yat-sen University, Guangzhou, China; 3 School of Public Health, Southern Medical University, Guangzhou, China; 4 Medical Big Data Center, Guangdong Provincial People's Hospital, Guangdong Academy of Medical Sciences, Southern Medical University, Guangzhou, China; 5 Guangdong Provincial Key Laboratory of Artificial Intelligence in Medical Image Analysis and Application, Guangzhou, China

**Keywords:** patent ductus arteriosus, twins, premature infant, ibuprofen, risk factor

## Abstract

**Background:**

Premature twins face a dual challenge: a high incidence of hemodynamically significant patent ductus arteriosus (hsPDA) and a poor response to ibuprofen. Current management, derived from singleton data, fails to address this population's unique risks. Our study specifically investigates risk factors for hsPDA development and ibuprofen treatment failure in preterm twins to enable personalized prevention and therapy. Thus, the potential biomarkers for the occurrence of hsPDA and ibuprofen closure failure were examined.

**Methods:**

A single-center, retrospective case-control study. 736 twin infants born at ≤34 weeks of gestation were included, of which 70 were exposed to hsPDA and 66 were exposed to ibuprofen. Clinical data were obtained from the Electronic Medical Record system. For the occurrence of hsPDA and failure of ibuprofen treatment, multivariate logistic regression was employed to identify the independent risk factor and the potential biomarkers were examined.

**Results:**

1. When compared to the non-hsPDA group, the hsPDA group demonstrated markedly higher incidences of low birth weight, lower gestational age, male gender, low 5 min Apgar scores, selective intrauterine growth restriction (sIUGR), use of pulmonary surfactant, respiratory distress syndrome (RDS), ≥grade III intraventricular hemorrhage, and early-onset sepsis (*p* < 0.05). The hsPDA group had a notably higher rate of sIUGR (28.6%) relative to the non-hsPDA group (15.2%, *p* = 0.004). Furthermore, in twins with sIUGR, the larger infant exhibited a higher hsPDA incidence than the non-hsPDA group (*p* < 0.05). 2. Univariate analysis revealed that vaginal delivery, birth weight, gestational age, male gender, 5 min Apgar score, early-onset sepsis, RDS, and sIUGR were associated with the occurrence of hsPDA (*p* < 0.05). Multivariate regression identified sIUGR [odds ratio (OR) = 3.337, 95% confidence interval (95% CI) 1.301–8.560] as an independent risk factor (IRF), while a higher birth weight (OR = 0.537, 95% CI 0.437–0.660) was noted to be a protective factor (*p* < 0.05). 3. Compared with the successful treatment group, the treatment failure group showed higher rates of premature rupture of membranes (PROMs) > 18 h, postnatal surfactant use, and monochorionic diamniotic (MCDA) twins (*p* < 0.0167, Bonferroni correction). The treatment failure group included 11 MCDA cases (73.3%), markedly more than the successful treatment group (33.3%, *p* = 0.006). 4. Univariate regression analysis identified gestational age, birth weight, MCDA, 5 min Apgar score, and PROMs > 18 h as factors associated with ibuprofen treatment failure (*p* < 0.05). Multivariate regression revealed MCDA (OR = 4.686, 95% CI 1.070–20.530) and PROMs > 18 h (OR = 15.198, 95% CI 2.377–97.178) as IRFs for ibuprofen treatment failure (*p* < 0.05). 5. In the pharmacological closure group, MCDA twins exhibited markedly lower closure rates after both the first and total two courses compared to dichorionic diamniotic twins (*p* < 0.05). MCDA twins required higher cumulative drug doses (40.21 ± 1.27 vs. 32.21 ± 0.53, *p* < 0.05), although no significant difference in the frequency of administration was detected. The MCDA group also had a higher rate of surgical interventions (39.29% vs. 10.53%, *p* < 0.05). 6. The NT-proBNP, Hs-cTn, PGE2, Cortisol, and TXA2 levels in the sIUGR group were markedly elevated versus those in the non-sIUGR group, with statistically significant differences (*p* < 0.05).

**Conclusions:**

In preterm twins ≤34 weeks, sIUGR is an IRF for hsPDA. In addition, MCDA and PROMs > 18 h are IRFs for ibuprofen treatment failure. Meanwhile, elevated levels of NT-proBNP and Hs-cTn may play a role in the development of hsPDA in preterm infants with sIUGR.

## Introduction

Hemodynamically significant patent ductus arteriosus (hsPDA) represents a common and clinically consequential complication in premature infants, typically presenting with systemic hypoperfusion and pulmonary overcirculation—both of which substantially influence clinical outcomes. In recent years, the widespread adoption of assisted reproductive technologies and advances in perinatal medicine have contributed to a growing number of twin pregnancies and preterm twin births, which in turn has been paralleled by an increased occurrence of hsPDA in this population (1, 2). Evidence suggests that the unique clinical features of preterm twins lead to distinct pathophysiological pathways, differing considerably from those observed in singletons both during gestation and after birth. Indeed, twin pregnancies are associated with markedly elevated risks of preterm delivery and neonatal complications, with perinatal mortality rates reported to be up to six times higher than in singleton pregnancies (3, 4). Importantly, the incidence of patent ductus arteriosus (PDA) among twins is four times that of singletons (5), while the rate of hsPDA is 1.41-fold greater (6). Compounding this clinical challenge, preterm twins with hsPDA demonstrate reduced responsiveness to cyclooxygenase inhibitor therapy (7).

Currently, pharmacologic intervention remains the cornerstone of hsPDA management. Ibuprofen, as a prostaglandin synthase inhibitor, lowers prostaglandin concentrations and induces ductal smooth muscle contraction, thereby reducing the aortopulmonary pressure gradient and promoting ductal closure. While previous studies have documented the incidence and treatment outcomes of hsPDA in both singleton and multiple preterm births, there remains a notable gap in research focused specifically on risk factors underlying hsPDA development and ibuprofen treatment failure in preterm twins. Given the distinctive pathophysiology characterizing this group, a systematic effort to identify twin-specific risk factors is essential to inform and refine clinical management.

This study is designed to progress through three logically sequential phases: firstly, to identify risk factors associated with hsPDA development in preterm twins ≤34 weeks' gestation; secondly, to elucidate factors contributing to ibuprofen treatment failure; and thirdly, to explore novel biological markers linked to both hsPDA occurrence and drug response, thereby translating clinical observations into potential mechanistic insights and future therapeutic avenues.

## Methods

### Participants

Clinical data from premature twins born at ≤34 weeks of gestation and admitted to the NICUs of Guangzhou Women and Children's Medical Center, Guangzhou Medical University, between January 2019 and June 2024, were retrospectively analyzed. Exclusion criteria included: intrauterine demise of either twin; congenital heart disease (excluding PDA and patent foramen ovale); life-threatening congenital defects or chromosomal/genetic abnormalities; and incomplete clinical data.

Biomarkers linked to the occurrence and pharmacological closure of hsPDA include N-terminal pro-brain natriuretic peptide (NT-proBNP), high-sensitivity cardiac troponin (Hs-cTn), prostaglandin E2 (PGE2), cortisol, thromboxane A2 (TXA2), platelets (PLT), plateletcrit (PCT), platelet distribution width (PDW), and mean platelet volume (MPV). The present biomarker study included a total of 48 neonates from a consecutively enrolled cohort at our hospital. This sample size constituted the total number of eligible cases available during the study period. Subjects were naturally allocated into sIUGR group, non-sIUGR group, and sIUGR + hsPDA group based on their confirmed clinical diagnoses of sIUGR and hsPDA. Blood samples (0.5 mL) were procured from the premature twins within 2 h after birth and placed in vacuum-dried blood collection tubes. The enzyme-linked immunosorbent assay was executed for detection.

The study protocol was approved by the Guangzhou Women and Children's Medical Center.

Institutional Ethics Committee (JBGS2024-11) with a waiver of informed consent.

### Study definitions

sIUGR in twins was defined as (8): (1) birth weight discordance ≥25% between twins; (2) the smaller twin's birth weight falling below the 10th percentile. Larger twin: the heavier twin. Smaller twin: the lighter twin. The determination of chorionicity was established by first-trimester ultrasound as the primary method, with definitive confirmation via postnatal placental pathological examination. Diagnostic criteria for hsPD A (9) included hemodynamic changes 72 h after birth, ductus arteriosus diameter >1.5 mm, along with one or more of the following clinical manifestations: cardiac murmur, tachycardia, tachypnea, increased pulse pressure, hypotension, water-hammer pulse, or cardiomegaly. Based on these criteria, subjects were categorized into hsPDA and non-hsPDA groups. In the hsPDA group, ibuprofen treatment was administered after excluding contraindications. Treatment failure was defined as persistent PDA following two courses (6 doses) of ibuprofen (10), and these cases were further divided into successful and failed treatment groups. The ibuprofen dosing data were retrieved from the electronic medical record system. The interval for each course followed the standard regimen (24 h intervals). Surgical intervention was considered for cases with persistent PDA after two courses of ibuprofen.

### Statistical analysis

Statistical analyses were conducted utilizing SPSS version 26.0. Categorical variables were expressed as frequencies (percentages, %) and compared across groups with the *χ*² test. When the assumptions for the *χ*² test were not satisfied, Fisher's exact test was utilized. The Kolmogorov–Smirnov test was employed to assess the normality of continuous variables. For normally distributed data, results were denoted as means ± standard deviation $\left(\bar{\chi }\pm s\right)$ and comparisons between groups were executed utilizing the *t*-test. For non-normally distributed data, non-parametric tests, such as the Mann–Whitney *U* test, were used. In subgroup analyses, a statistical power analysis was performed to evaluate the adequacy of sample sizes, and a Bonferroni correction was applied, adjusting the α level to 0.0167. Univariate analysis was performed for all variables, and those demonstrating statistical significance were subsequently included in multivariate logistic regression analysis. Statistical significance was set at *p* < 0.05.

## Results

**Figure 1:**
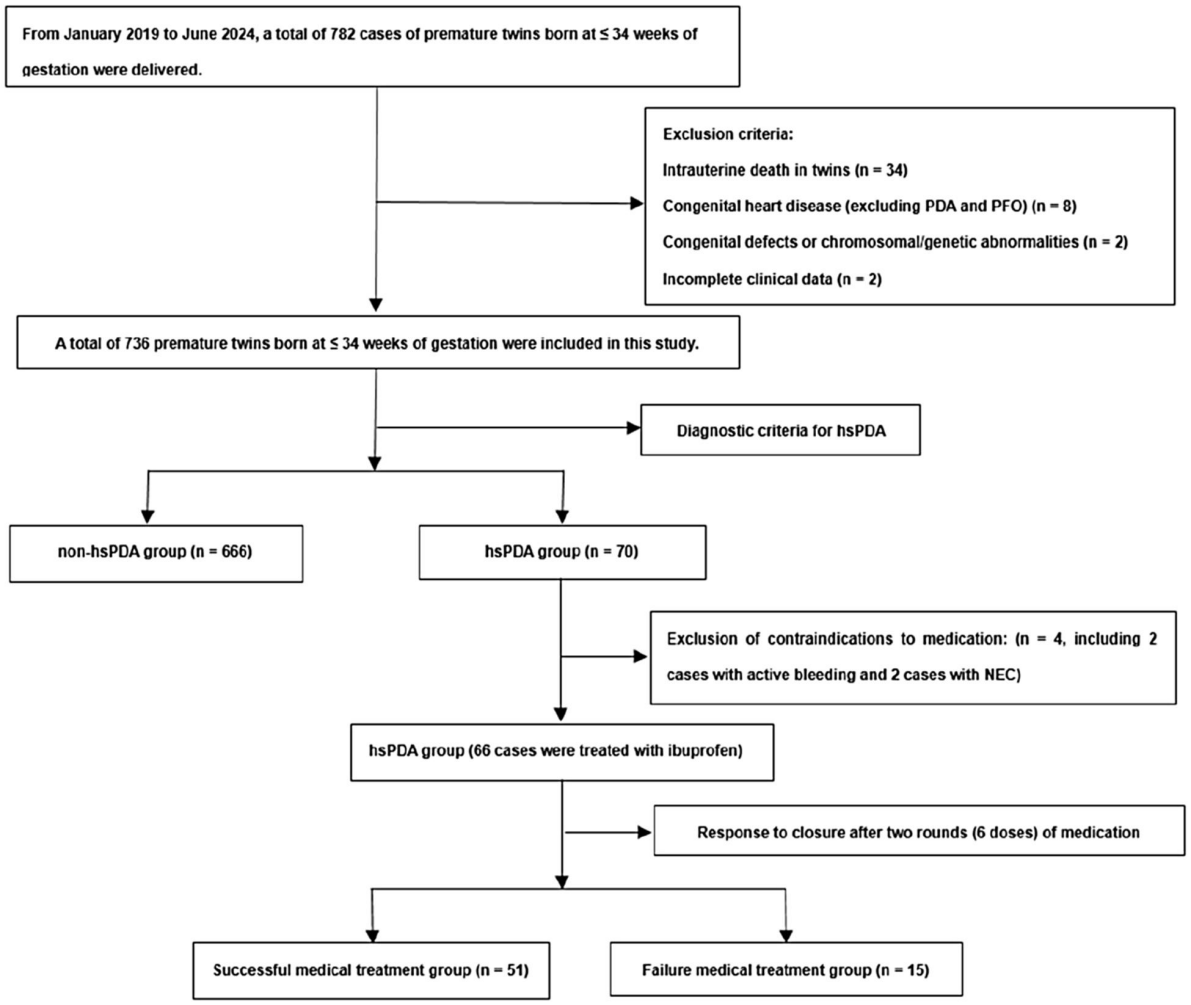
Flowchart of study population. hsPDA, hemodynamically significant patent ductus arteriosus; NEC, necrotizing enterocolitis; PFO, patent foramen ovale.

A sum of 736 twin infants born at ≤34 weeks of gestation were included in this investigation, 70 cases were diagnosed with hsPDA. Among these, 66 cases were treated with ibuprofen, while 4 cases were managed with either conservative medical therapy or surgical intervention due to contraindications to using ibuprofen. Closure was achieved in 51 cases after one to two courses of ibuprofen, whereas 15 cases required surgical intervention following the failure of two courses of medical treatment. All 15 cases who underwent surgical ligation achieved successful outcomes, with confirmed complete anatomical closure of the ductus postoperatively (Figure 1).

An analysis of maternal factors between the two groups revealed that only the mode of delivery exhibited statistical significance, with the hsPDA group displaying a notably higher rate of vaginal delivery than the non-hsPDA group (*p* < 0.05) (Table 1).

**Table 1:** General characteristics of premature twins in hsPDA group and non-hsPDA group.

Variables	hsPDA	Non-hsPDA	*p*-value
	(*n* = 70)	(*n* = 666)	
Maternal factors			
Maternal age, years, mean ± SD	30.8 ± 5.1	31.5 ± 4.6	0.222
Vaginal delivery, *n* (%)	14 (20.0)	68 (10.2)	0.013
HDP, *n* (%)	8 (11.4)	78 (11.7)	0.944
GDM, *n* (%)	12 (17.1)	125 (18.8)	0.740
PROMs >18 h, *n* (%)	9 (12.9)	98 (14.7)	0.675
Placental abruption, *n* (%)	3 (4.3)	22 (3.3)	0.724*
Complete course of dexamethasone, *n* (%)	40 (57.1)	442 (66.4)	0.123
Magnesium sulfate, *n* (%)	20 (10.6)	169 (25.4)	0.560
NSAIDS, *n* (%)	3 (4.3)	27 (4.1)	0.758*
MCDA, *n* (%)	29 (41.4)	251 (37.7)	0.540
DCDA, *n* (%)	41 (58.6)	387 (58.1)	0.728
MCMA, *n* (%)	0	28 (4.2)	0.099*
sIUGR, *n* (%)	20 (28.6)	102 (15.3)	0.005
Larger twin with sIUGR, *n* (%)	10 (14.3)	50 (7.5)	0.049
Smaller twin with sIUGR, *n* (%)	10 (14.3)	52 (7.8)	0.063
TTTS, *n* (%)	9 (31)	41 (16.3)	0.050
Neonatal factors			
GA, weeks, mean ± SD	29.4 ± 2.2	32.1 ± 1.7	<0.001
BW, g, mean ± SD	1,179 ± 359	1,643 ± 365	<0.001
Larger twin, *n* (%)	37 (52.9)	331 (49.7)	0.615
Smaller twin, *n* (%)	33 (47.1)	335 (50.3)	0.615
First delivered, *n* (%)	28 (40.0)	340 (51.5)	0.079
Gender (male), *n* (%)	28 (40.0)	365 (54.8)	0.018
IVF, *n* (%)	24 (34.3)	231 (34.7)	0.947
5 min Apgar score, mean ± SD	8.7 ± 0.9	9.2 ± 0.8	<0.001
RDS, *n* (%)	59 (84.3)	392 (58.9)	<0.001
IVH ≥ grade III, *n* (%)	6 (8.6)	5 (0.8)	0.001*
EOS, *n* (%)	6 (8.8)	5 (0.8)	<0.001*
PS administration, *n* (%)	46 (65.7)	112 (16.8)	<0.001

* Fisher's exact test; BW, birth weight; DCDA, dichorionic diamniotic; EOS, early-onset sepsis; GA, gestational age; GDM, gestational diabetes mellitus; HDP, hypertension disorders of pregnancy; hsPDA, hemodynamically significant patent ductus arteriosus; IVF, *in vitro* fertilization; MCDA, monochorionic diamniotic; MCMA, monochorionic monoamniotic; NSAIDS, non-steroidal anti-inflammatory drugs; PROMs, premature rupture of membranes; PS, pulmonary surfactant; RDS, respiratory distress syndrome; ICH, intraventricular hemorrhage; sIUGR, selective intrauterine growth restriction; TTTS, twin-to-twin transfusion syndrome.

A comparison of the basic characteristics between the two groups revealed that the gestational age (GA) and birth weight (BW) in the hsPDA group were notably lower than those in the non-hsPDA group (*p* < 0.05). The proportions of sIUGR and TTTS in the hsPDA group were markedly higher than in the non-hsPDA group (*p* < 0.05). Furthermore, a lower proportion of male infants and lower 5 min Apgar scores were observed in the hsPDA group in comparison to the non-hsPDA group. The incidence rates of neonatal complications, including respiratory distress syndrome (RDS), grade III or higher intraventricular hemorrhage (ICH), and early-onset sepsis (EOS), were markedly higher in the hsPDA group (*p* < 0.05). Given the inability to establish a clear temporal relationship between necrotizing enterocolitis, bronchopulmonary dysplasia, retinopathy of prematurity, and hsPDA, these factors were excluded from the multivariate analysis (Table 1).

The univariate analysis indicated that factors such as mode of delivery, birth weight, gestational age, gender, 5-minute Apgar score, early-onset sepsis, NRDS, and sIUGR were markedly correlated with the incidence of hsPDA in premature twins (*p* < 0.05). Multivariate logistic regression analysis identified sIUGR [odds ratio (OR) = 3.337, 95% confidence interval (95% CI) 1.301–8.560] as an independent risk factor (IRF) for hsPDA in premature twins (*p* < 0.05), while an increased GA(OR = 0.537, 95% CI 0.437–0.660) was noted to be an independent protective factor against hsPDA in premature twins (*p* < 0.05) (Table 2).

**Table 2:** Univariate and multivariate analysis of risk factors for hsPDA in premature twins.

Variables	Univariate analysis		Multivariate analysis	
	OR (95% CI)	*p*-value	OR (95% CI)	*p*-value
Mode of delivery (vaginal)	2.199 (1.163–4.157)	0.015		
BW	0.997 (0.996–0.998)	<0.001		
GA	0.554 (0.489–0.627)	<0.001	0.537 (0.437–0.660)	<0.001
Gender (male)	0.550 (0.333–0.908)	0.019		
5 min Apgar score	0.568 (0.447–0.722)	<0.001		
EOS	12.394 (3.680–41.739)	<0.001		
RDS	3.749 (1.934–7.268)	<0.001		
sIUGR	2.238 (1.278–3.918)	0.005	3.337 (1.301–8.560)	0.012

BW, birth weight; GA, gestational age; EOS, early-onset sepsis; hsPDA, hemodynamically significant patent ductus arteriosus; RDS, respiratory distress syndrome; sIUGR, selective intrauterine growth restriction.

In the successful treatment group, the incidence of premature rupture of membranes (PROMs) > 18 h in mothers was notably lower compared to the treatment failure group (*p* < 0.0167) (Table 3).

**Table 3:** General characteristics of premature twins with successful and failed ibuprofen treatment for hsPDA.

Variables	Successful ibuprofen treatment group	Failed ibuprofen treatment group	*p*-value
	(*n* = 51)	(*n* = 15)	
Maternal factors			
Maternal age, years, mean ± SD	30.6 ± 5.1	32.0 ± 4.5	0.364
Vaginal delivery, *n* (%)	10 (19.6)	4 (26.7)	0.720*
HDP, *n* (%)	7 (13.7)	0	0.336*
GDM, *n* (%)	6 (11.8)	4 (26.7)	0.217*
PROMs > 18 h, *n* (%)	3 (5.9)	5 (33.3)	0.012*
Complete course of dexamethasone, *n* (%)	29 (56.9)	7 (46.7)	0.486
Magnesium sulfate, *n* (%)	14 (27.5)	3 (20.0)	0.742*
NSAIDS, *n* (%)	3 (5.9)	0	1.000*
DCDA, *n* (%)	34 (66.7)	4 (26.7)	0.006
MCDA, *n* (%)	17 (33.3)	11 (73.3)	0.006
sIUGR, *n* (%)	15 (29.4)	2 (13.3)	0.322*
TTTS, *n* (%)	4 (7.8)	4 (26.7)	0.071*
Neonatal factors			
GA, weeks, mean ± SD	29.7 ± 2.1	28.2 ± 2.1	0.025
BW, g, mean ± SD	1,250 ± 344	1,007 ± 327	0.018
Gender (male), *n* (%)	20 (39.2)	5 (33.3)	0.680
IVF, *n* (%)	20 (39.2)	4 (26.7)	0.374*
5 min Apgar score, mean ± SD	8.8 ± 0.7	8.3 ± 0.9	0.025
RDS, *n* (%)	42 (82.4)	13 (86.7)	1.000
IVH ≥ Grade III, *n* (%)	4 (7.8)	2 (13.3)	0.612*
EOS, *n* (%)	4 (7.8)	1 (6.7)	1.000*
PS administration, *n* (%)	32 (62.7)	4 (26.7)	0.014*

* Fisher's exact test; BW, birth weight; DCDA, dichorionic diamniotic; EOS, early-onset sepsis; GA, gestational age; GDM, gestational diabetes mellitus; HDP, hypertension disorders of pregnancy; hsPDA, hemodynamically significant patent ductus arteriosus; ICH, intraventricular hemorrhage; IVF, *in vitro* fertilization; MCDA, monochorionic diamniotic; MCMA, monochorionic monoamniotic; NSAIDS, non-steroidal anti-inflammatory drugs; PROMs, premature rupture of membranes; PS, pulmonary surfactant; RDS, respiratory distress syndrome; sIUGR, selective intrauterine growth restriction; TTTS, twin-to-twin transfusion syndrome.

The univariate analysis of the two groups suggested that birth weight, gestational age, MCDA, 5 min Apgar score, and PROMs > 18 h were markedly associated with ibuprofen treatment failure in premature twins with hsPDA (*p* < 0.05). Multivariate logistic regression analysis of these variables identified MCDA (OR = 4.686, 95% CI 1.070–20.530) and PROMs > 18 h (OR = 15.198, 95% CI 2.377–97.178) as IRFs for ibuprofen treatment failure in premature twins with hsPDA (*p* < 0.05) (Table 4).

**Table 4:** Univariate and multivariate analysis of ibuprofen treatment failure in premature twins with hsPDA.

Variables	Univariate analysis		Multivariate analysis	
	OR (95% CI)	*p*-value	OR (95% CI)	*p*-value
GA	0.726 (0.542–0.972)	0.031		
BW	0.998 (0.996–1.000)	0.025		
MCDA	4.000 (1.179–13.565)	0.026	4.686 (1.070–20.530)	0.040
5 min Apgar score	0.475 (0.236–0.957)	0.037		
PROMs > 18 h	8.000 (1.639–39.043)	0.010	15.198 (2.377–97.178)	0.004

BW, birth weight; GA, gestational age; hsPDA, hemodynamically significant patent ductus arteriosus; MCDA, monochorionic diamniotic; PROMs, premature rupture of membranes.

Within the successful treatment group, a higher proportion of dichorionic diamniotic (DCDA) twins was observed. Among MCDA twins, the success rate of medical treatment was markedly lower than that in the treatment failure group (*p* < 0.0167) (Table 3). In the group receiving successful medical treatment, markedly lower closure rates were observed in MCDA compared to DCDA, both after the first course and across the two courses of treatment. Higher doses of ibuprofen were required for MCDA (*p* < 0.05), though no significant differences were found in the frequency of medication administration. Furthermore, a markedly higher rate of surgical intervention was noted in MCDA (*p* < 0.05) (Table 5).

**Table 5:** Comparison of medication usage among different types of twins.

Variables	MCDA group	DCDA group	*p*-value
	(*n* = 28)	(*n* = 38)	
Successful medical treatment group			
Closure after first course, *n* (%)	14 (50.0)	28 (73.6)	0.048
Closure after first and second courses, *n* (%)	17 (60.7)	34 (89.4)	0.006
Drug dosage (mg/kg), mean ± SD	40.2 ± 1.2	32.2 ± 0.5	<0.001
Number of doses administered, mean ± SD	3.5 ± 0.2	3.2 ± 0.2	0.472
Medical treatment failure group			
Surgical intervention, *n* (%)	11 (39.3)	4 (10.5)	0.006*

* Fisher's exact test; DCDA, dichorionic diamniotic; MCDA, monochorionic diamniotic.

The NT-proBNP, Hs-cTn, PGE2, Cortisol, TXA2, and PLT levels in the sIUGR with hsPDA group were markedly elevated in comparison to both the sIUGR and non-sIUGR groups (*p* < 0.05). Among the cases without hsPDA, the sIUGR group exhibited notably higher concentrations of NT-proBNP and Hs-cTn when compared to the non-sIUGR group (*p* < 0.05). Concerning the indicators of drug intervention efficacy, the sIUGR group demonstrated markedly greater levels of PGE2, TXA2, and Cortisol than the non-sIUGR group (*p* < 0.05) (Table 6).

**Table 6:** Analysis of biological indicators among sIUGR group, non-sIUGR group, and sIUGR + hsPDA group.

Laboratory findings	sIUGR group	non-sIUGR group	sIUGR + hsPDA group	*p1*-value	*p2*-value	*p3*-value	*p*-value
	(*n* = 16)	(*n* = 26)	(*n* = 6)				
PGE2, pg/mL	612.46 ± 67.37	397.49 ± 47.89	859.90 ± 16.20	<0.001	<0.001	<0.001	<0.001
NT-proBNP, pg/mL	339.43 ± 32.92	195.43 ± 31.41	462.70 ± 35.83	<0.001	<0.001	<0.001	<0.001
Cortisol, nmol/L	1,127.99 ± 128.47	723.93 ± 120.18	1,623.15 ± 102.67	<0.001	<0.001	<0.001	<0.001
TXA2, pg/mL	214.53 ± 20.94	135.17 ± 18.24	289.78 ± 16.69	<0.001	<0.001	<0.001	<0.001
Hs-cTn, mg/L	14.88 ± 1.76	8.57 ± 1.78	21.82 ± 0.64	<0.001	<0.001	<0.001	<0.001
PLT, *10^9^/L	227.75 ± 61.42	235.62 ± 53.10	157.17 ± 27.32	0.663	0.029	0.004	0.015
PCT, %	0.21 ± 0.05	0.22 ± 0.05	0.17 ± 0.03	0.495	0.181	0.054	0.140
PDW, %	9.67 ± 1.60	9.78 ± 1.02	10.70 ± 0.39	0.780	0.189	0.073	0.265
MPV, fl	9.61 ± 0.89	9.59 ± 0.48	9.98 ± 0.32	0.939	0.415	0.148	0.505

*P1*-value: comparison between sIUGR group and non-sIUGR group; *P2*-value: comparison between sIUGR group and sIUGR + hsPDA group; *P3*-value: comparison between non-sIUGR group and sIUGR + hsPDA group; *P-value*: comparison among the three groups. A *P*-value < 0.05 was considered statistically significant.

PGE2, prostaglandin E2; NT-proBNP, nterminal pro-brain natriuretic peptide; TXA2, thromboxane A2; Hs-cTn, hypersensitive troponinI; PLT, platelet count; PCT, plateletcrit; PDW, platelet distribution width; MPV, mean platelet volume; hsPDA, hemodynamically significant patent ductus arteriosus; sIUGR, selective intrauterine growth restriction.

## Discussion

HsPDA, results in systemic hypoperfusion and pulmonary congestion, thereby raising the likelihood of complications such as necrotizing enterocolitis, ICH, bronchopulmonary dysplasia, and retinopathy of prematurity, ultimately contributing to elevated mortality rates (11, 12). Previous research has demonstrated that premature twins experience a higher incidence of hsPDA than singleton infants (2), with diminished sensitivity to cyclooxygenase inhibitors, although these studies were constrained by small sample sizes and ambiguous twin classifications (3). Furthermore, no prior investigations have reported whether risk factor for hsPDA in twins differ from those in singletons. Preterm twin gestation, characterized by distinct pathophysiology and complications such as TTTs and sIUGR, necessitates the identification of unique risk factors to inform and optimize clinical management strategies.

Numerous studies have established that factors such as lower GA, reduced BW, prenatal hormonal influences, birth asphyxia, RDS, small for gestational age, and intrauterine infections are linked to the occurrence of hsPDA in singleton premature infants (13, 14). However, the intrauterine environment in twins differs markedly from that of singletons, resulting in distinct disease patterns. Ferraz et al. (15) reported a higher prevalence of PDA in MCDA twins compared to DCDA twins. Yamaguchi et al. (16) found that MCDA twins exhibited markedly higher hsPDA rates than singletons. Another investigation (17) found no statistically significant differences in PDA incidence among different types of twin pregnancies. In contrast, the current study identified no significant differences in hsPDA rates between MCDA and DCDA twins.

The current study identified highlighted sIUGR as a significant risk factor for hsPDA in twins. Similar findings were reported by Yang CY's team (18). The present study also demonstrated that in sIUGR twin pairs, the larger infant had a markedly higher incidence of hsPDA (*P* < 0.05). Additional studies (19) have shown increased PDA rates in twins with weight discordance, particularly when weight differences exceeded 30%, with the larger twin showing a higher incidence of PDA. This observation aligns with the current results, which showed a higher hsPDA incidence in the larger infant of sIUGR twin pairs. In this investigation, consistent with several singleton studies, increased gestational age was confirmed as a protective factor against hsPDA. This may be attributed to the fact that a lower GA is associated with less mature ductus arteriosus smooth muscle and weaker elastic fibers in the intima, thereby preventing the development of abundant intimal cushions (20), which leads to delayed ductal closure and an increased incidence of hsPDA.

Given the persistent debates surrounding the selection of medications for the treatment of hsPDA, a consensus has yet to be established, although pharmacological intervention is still regarded as the first-line approach (21). Ibuprofen has increasingly supplanted indomethacin owing to the latter's extensive list of adverse effects (22). Consequently, ibuprofen was adopted as the primary pharmacological therapy at our center, and all participants in this investigation were administered ibuprofen.

Numerous investigations have suggested that the closure rate of ibuprofen treatment in preterm infants ranges from 55% to 89% (10, 23–25). In this investigation, the success rate of ibuprofen closure in premature twins was observed to be 77.3%. A study by Utsumi M (26) found that twins exhibited a markedly higher failure rate in ibuprofen treatment compared to singletons (39% vs. 75%), multiple pregnancies were identified as IRF for ibuprofen treatment failure in preterm infants with hsPDA. In the current study, MCDA and PROMs > 18 h were identified as IRF for ibuprofen treatment failure in preterm twins with hsPDA (*p* < 0.05). Research has shown that the closure of the ductus arteriosus is influenced by intimal thickening (27). Ito et al. (28) used premature sheep models to demonstrate that twins exhibited less intimal thickening than singletons, offering an anatomical explanation for the increased susceptibility of twins to hsPDA and their reduced responsiveness to cyclooxygenase inhibitors. The intrauterine environment of twins, especially in MCDA twins with vascular anastomoses, differs markedly from that of singletons, and an unstable cardiovascular state is currently believed to facilitate the onset of hsPDA. However, large-scale studies investigating the differences in treatment response across various twin types remain lacking. DCDA twins responded favorably to ibuprofen treatment, while MCDA twins were found to be at a markedly higher risk of treatment failure, with the underlying mechanisms requiring further investigation. Whether prophylactic intervention or medication adjustment is necessary for MCDA twin pregnancies remains a clinically relevant question worthy of further investigation, which also highlights a clear direction for further research. We agree that the limited number of events (70 hsPDA, 15 treatment failures) may increase the risk of model overfitting. As this was a retrospective study, the current dataset represents the maximum available sample under existing conditions. To mitigate this limitation, we minimized the number of covariates entered into the multivariate logistic regression model—only variables that were statistically significant (*p* < 0.05) in univariate analysis and considered clinically relevant were included, thereby controlling model complexity. Future studies with larger sample sizes and validation analyses (e.g., bootstrapping or penalized regression) are planned to further confirm the robustness of our findings. The wide confidence interval for the “PROMs ≥ 18 h” subgroup reflects instability of the risk estimate, mainly due to the small number of cases in this subgroup and the resulting sparse data distribution. To assess robustness, we rechecked the univariate and multivariate models, and the direction of association remained consistent, supporting the validity of the observed trend. Nevertheless, the high point estimate suggests that prolonged PROMs may be an important clinical risk factor, warranting confirmation in future prospective or larger multicenter studies.

According to the findings by Olgun et al. (10), the closure rates of hsPDA in preterm infants after the first, second, and third courses of oral ibuprofen treatment were 71%, 83%, and 88%, respectively. Compared with the first course, the second course resulted in a statistically significant increase in the ductal closure rate (*p* < 0.05), while no significant difference was observed between the third and second courses (*p* > 0.05). Data from the Ono T tea m^7^ indicated that twin pregnancies with hsPDA required a higher median dose of cyclooxygenase inhibitors and had a greater need for surgical ligation compared to singletons [5 vs. 2 (*p* < 0.001) and 42% vs. 21% (*p* = 0.018), respectively], suggesting a lower sensitivity to pharmacological treatment in twins. Our study further revealed that MCDA twins had significantly lower closure rates than DCDA twins after the first course and overall after two courses (50.00% vs. 73.68%; 60.71% vs. 89.47%, *p* < 0.05). Additionally, MCDA twins required a higher total drug dose (40.21 ± 1.27 vs. 32.21 ± 0.53, *p* < 0.05), although no significant difference was found in the number of drug administrations. Moreover, the MCDA group showed a significantly higher rate of surgical intervention (39.29% vs. 10.53%, *p* < 0.05). These results suggest that among preterm twins with hsPDA, MCDA twins require higher medication doses and are more likely to undergo surgical intervention.

Recent clinical investigations have indicated a correlation between serum concentrations of NT-proBNP, Hs-cTn, PLT, PCT, PDW, and MPV with the onset of hsPDA (29, 30). Additionally, the serum concentrations of PGE2, TXA2, Cortisol, PLT, PCT, PDW, and MPV have been linked to the effectiveness of pharmacological interventions (31). In the present study, the levels of NT-proBNP and Hs-cTn were markedly elevated in the sIUGR group compared to the non-sIUGR group. This observation aligns with the findings of Fujioka K et al. (32, 33), who reported increased NT-proBNP levels in sIUGR premature twins. Similarly, Bahlmann et al. (34) observed that, in comparison to appropriate for gestational infants, IUGR fetuses exhibited substantially higher NT-proBNP concentrations in umbilical cord blood (35). The elevated NT-proBNP levels in sIUGR premature twins may be associated with cardiac overload resulting from renin-angiotensin system activation, hypoxemia due to reduced blood volume, and intrauterine growth restriction (36, 37). Moreover, this study revealed, for the first time, markedly increased Hs-cTn concentrations in the sIUGR group. Although the underlying cause of this elevation warrants further exploration, it is hypothesized that both NT-proBNP and Hs-cTn are implicated in the pathogenesis of hsPDA in sIUGR premature twins. The sIUGR + hsPDA group demonstrated significantly lower birth weights and gestational ages compared to other groups. Therefore, the observed dysregulation in biomarker profiles is likely attributable to the confounding effects of prematurity, fetal growth restriction, and shared underlying placental pathology, rather than representing a distinct, condition-specific causal pathway. This interpretation, however, awaits validation in future prospective studies designed to control for these potential confounders.

SIUGR has been recognized as an IRF for hsPDA, although it does not appear to influence the failure of pharmacological interventions. This suggests that while premature twins with sIUGR are at an increased risk for developing hsPDA, this condition does not show a correlation with the success of pharmacological closure. Among the markers related to the efficacy of pharmacological intervention, the sIUGR group exhibited markedly elevated concentrations of PGE2, TXA2, and Cortisol compared to the non-sIUGR group (*p* < 0.05). In the study cohort, a higher proportion of the sIUGR group had received complete prenatal dexamethasone administration (Supplementary Table S1), which may account for the increased serum Cortisol concentrations. PGE2 is known to play a pivotal role in sustaining ductal patency, whereas TXA2 acts as a potent inducer of platelet activation. Platelets contribute to ductal closure by adhering to the contracted ductal wall, recruiting additional circulating platelets, and promoting the formation of an intraluminal thrombus (38). The opposing actions of TXA2 and PGE2 in the pharmacological closure of hsPDA, when considered together, may help explain why sIUGR does not markedly influence the outcomes of pharmacological treatment. The limited sample size (*n* = 48) restricts the statistical power for multi-analyte biomarker comparisons. Given this limitation, formal power analysis was not performed, as the primary aim of this component was exploratory—to identify potential biomarker signals and generate hypotheses for future validation. We therefore interpreted these findings based on effect sizes and confidence intervals rather than on statistical significance alone, to avoid over interpretation of underpowered results. Future large-scale studies will incorporate adequate sample size estimation and formal power analysis to confirm these preliminary findings.

This retrospective, single-center case-control study was subject to unavoidable selection bias, and given that all data were extracted from the medical record system, certain relevant indicators may have been omitted due to incomplete clinical documentation. Findings from single-center data can be subject to variations in patient baseline characteristics, which may compromise the generalizability of the conclusions. We acknowledge that this design may introduce selection bias and limit external validity due to differences in admission criteria, treatment, and follow-up. To mitigate these biases, we predefined strict inclusion and exclusion criteria and applied standardized diagnostic and therapeutic protocols. Multivariate logistic regression was used to adjust for key confounders such as gestational age, birth weight, and sIUGR. Nevertheless, residual confounding cannot be completely excluded. Future multicenter prospective studies using propensity score matching are planned to further enhance the robustness and generalizability of our findings. We adopted the definition of treatment failure as “persistent PDA after two consecutive courses of ibuprofen treatment,” an operational definition widely used in multiple similar studies due to its clarity and ease of standardization. However, this definition has certain limitations, as it fails to fully account for factors such as the dose-response relationship, retreatment intervals, and individual variations in plasma drug concentrations. These aspects will be addressed as important directions for in-depth exploration and refinement in the design and analysis of future prospective studies. With regard to the detection of sIUGR biological indicators, only data from a subset of study subjects were included. To address potential inaccuracies in the findings, the Bonferroni correction was employed for multiple comparisons during statistical analysis.

## Conclusion

The study confirmed that sIUGR is an IRF for hsPDA in premature twins, while higher GA at birth serves as a protective factor. In addition, MCDA pregnancies and PROM > 18 h were identified as IRFs for ibuprofen treatment failure in twins with hsPDA. Elevated NT-proBNP and Hs-cTn levels may also contribute to hsPDA development in preterm infants with sIUGR, indicating their potential utility as biomarkers for further study. These findings highlight the importance of closely monitoring sIUGR twins for hsPDA, personalizing ibuprofen therapy based on pregnancy type and PROM duration, and exploring the role of NT-proBNP and Hs-cTn in early risk stratification and management of hsPDA in this vulnerable population.

## Data Availability

The original contributions presented in the study are included in the article/Supplementary Material, further inquiries can be directed to the corresponding author.
