# Supplementary material for: Risk factors for hemodynamically significant patent ductus arteriosus and ibuprofen treatment failure in premature twins: a retrospective case-control study
Source: Front Cardiovasc Med. 2026 Jan 12;12:1687361. doi: 10.3389/fcvm.2025.1687361 (PMC12832987; doi:10.3389/fcvm.2025.1687361)
Supplement: Supplementary file 1 [file Table1.docx]

Supplementary Table 1: Comparison of basic clinical data

|  | sIUGR group  (n＝16) | Non-sIUGR group (n＝26) | sIUGR+hsPDA group (n＝6) | *P1*-value | *P2*-value | *P3*-value | *P*-  value |
| --- | --- | --- | --- | --- | --- | --- | --- |
| Maternal factors |  |  |  |  |  |  |  |
| Maternal age, years, mean±SD | 29.8±3.3 | 30.6±3.2 | 29.3±3.6 | 0.484 | 0.734 | 0.396 | 0.616 |
| Vaginal delivery, *n* (%) | 2（12.5） | 4（15.4） | 3（50） | 0.047 | 0.814 | 0.053 | 0.112 |
| HDP, *n* (%) | 2（12.5） | 2（7.7） | 0 | 0.595 | 0.361 | 0.551 | 0.646 |
| GDM, *n* (%) | 6（37.5） | 6（23.1） | 0 | 0.298 | 0.076 | 0.244 | 0.193 |
| PROMs >18h, *n* (%) | 2（12.5） | 4（15.4） | 1（16.7） | 0.804 | 0.812 | 0.938 | 0.959 |
| Complete course of dexamethasone, *n* (%) | 14（87.5） | 14（53.8） | 6（100） | 0.017 | 0.544 | 0.021 | 0.014 |
| Magnesium sulfate, *n* (%) | 10（62.5） | 8（30.8） | 3（50） | 0.047 | 0.596 | 0.390 | 0.130 |
| Neonatal factors |  |  |  |  |  |  |  |
| GA, weeks, mean±SD | 32.0±1.8 | 32.1±2.2 | 29.9±1.9 | 0.841 | 0.044 | 0.024 | 0.071 |
| BW, g, mean±SD | 1522±424 | 1712±389 | 1306±325 | 0.138 | 0.260 | 0.028 | 0.059 |
| MCDA, *n* (%) | 10（62.5） | 14（53.8） | 6（100） | 0.570 | 0.107 | 0.038 | 0.113 |
| Male, *n* (%) | 10（62.5） | 14（53..8） | 3（50） | 0.596 | 0.611 | 0.869 | 0.824 |
| 5-min Apgar score, mean±SD | 9.3±0.6 | 9.7±0.8 | 8.8±1.3 | 0.184 | 0.189 | 0.023 | 0.057 |

*P1*-value: comparison between sIUGR group and non-sIUGR group; *P2*-value: comparison between sIUGR group and sIUGR+hsPDA group; *P3*-value: comparison between non-sIUGR group and sIUGR+hsPDA group; *P*-value: comparison among the three groups. A *P*-value < 0.05 was considered statistically significant.

hsPDA：hemodynamically significant patent ductus arteriosus；HDP：hypertension disorders of pregnancy;GDM：gestational diabetes mellitus;PROMs：premature rupture of membranes; GA：gestational age；BW：birth weight;MCDA：monochorionic diamniotic; sIUGR：selective intrauterine growth restriction
